# Meiosis through three centuries

**DOI:** 10.1007/s00412-024-00822-0

**Published:** 2024-05-10

**Authors:** Gareth Jones, Nancy Kleckner, Denise Zickler

**Affiliations:** 1 School of Biosciences, University of Birmingham, Birmingham, B15 2TT UK; 2 Department of Molecular and Cellular Biology, Harvard University, Cambridge, MA 02138 USA; 3 Institute for Integrative Biology of the Cell (I2BC), Centre National de La Recherche Scientifique (CNRS), Université Paris-Sud, Université Paris-Saclay, 91198 Gif-Sur-Yvette, France

**Keywords:** Meiosis, Crossing over, Pairing, Synaptonemal complex, Evolution

## Abstract

Meiosis is the specialized cellular program that underlies gamete formation for sexual reproduction. It is therefore not only interesting but also a fundamentally important subject for investigation. An especially attractive feature of this program is that many of the processes of special interest involve organized chromosomes, thus providing the possibility to see chromosomes "in action". Analysis of meiosis has also proven to be useful in discovering and understanding processes that are universal to all chromosomal programs. Here we provide an overview of the different historical moments when the gap between observation and understanding of mechanisms and/or roles for the new discovered molecules was bridged. This review reflects also the synergy of thinking and discussion among our three laboratories during the past several decades.

## Introduction: discovery and understanding of the meiotic process

The term “meiosis” (originally maiosis) was first proposed by Farmer and Moore (1905). To quote: “We think it desirable, in the interests of clarity, to explain the meaning of the nomenclature that is employed in this memoir in connection with the “reduction” divisions (sic)." This immediately tells us that by 1905 there was already a clear understanding that in the life cycles of sexually reproducing eukaryotes there is, associated with nuclear fertilization, a compensating process of nuclear/chromosomal reduction. Furthermore, it seems clear that by this time these authors understood that two consecutive nuclear divisions are involved in meiosis and also, contrary to earlier beliefs, that neither division is essentially, on its own, a reducing division.

**Fig. 1:**
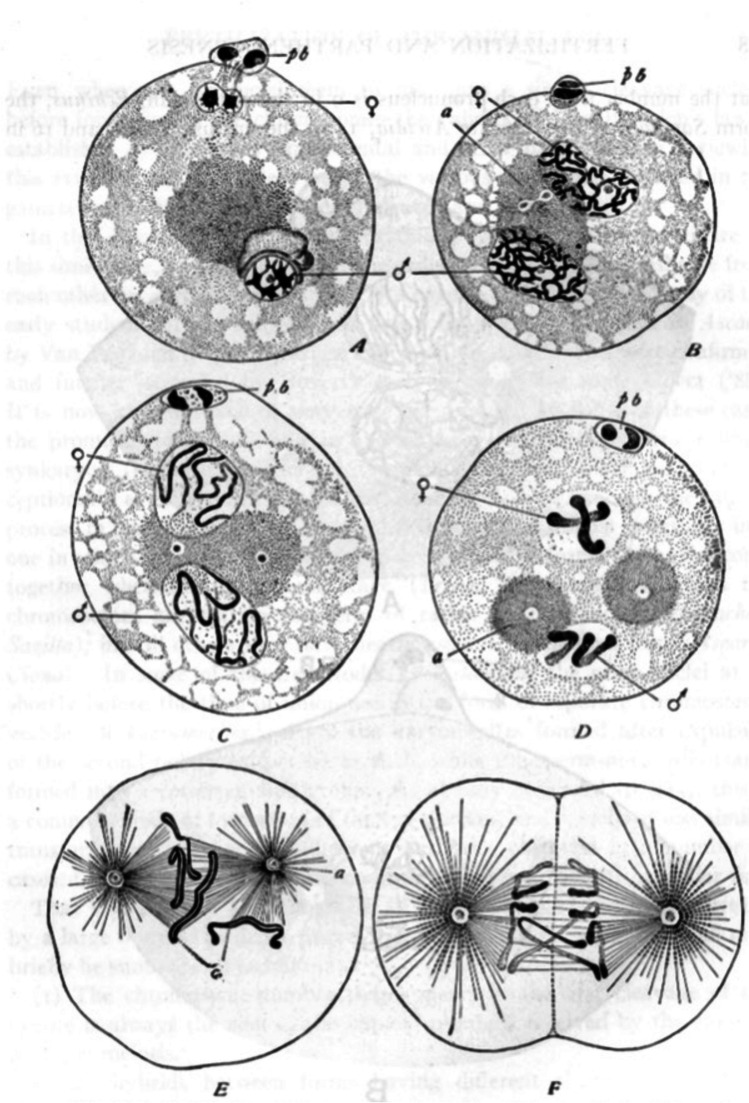
Demonstration by Boveri (1890; Fig. 197) that egg and sperm contribute equal numbers of chromosomes to the zygote during the fertilization of the egg of *Ascaris megalocephala*. Using the fact that this Ascaris has large clear cells and only two pairs of chromosomes, Boveri was able to trace the fate of egg and sperm chromosomes in cell lineages with great precision. This is illustrated here for the second meiotic division. **A** The sperm nucleus (shown by its symbol) has entered the oocyte (shown by its symbol); above is indicated the extrusion of the first polar body (pb), which occurs when meiosis I is achieved. **B** The oocyte enters meiosis II as indicated by the early prophase stage in the two (female and male) pronuclei. The grey sphere (a) indicates the central body. **C** Chromosomes are more compact in the pronuclei and the central body is divided. The second polar body (pb) has divided as indicated by the two polar dark masses. **D** The two sister chromatids are now visible and the central body is divided into two spheres (a). **E** and **F** correspond, respectively, to metaphase and anaphase of meiosis II. **F** Cleavage is in progress while daughter-chromosomes move towards the spindle-poles. The drawings illustrate, for the first time, that chromosome numbers are reduced in half when they enter meiosis II and that male and female nuclei provide the same number of chromosomes

However, to comprehend the tortuous path that led to this very basic understanding of the meiotic process it is necessary to go back at least 25 years to the period of early cytological studies into cell division and reproductive processes and in particular to the maturation of animal eggs. This focus on animal eggs for cytological study, especially in the early 1880s, was perhaps ascribable to the large size of oocytes compared to other cells, considering the relatively basic microscopy that was available during that era. The most important observations and conclusions from these early studies were, firstly, that egg and sperm contributed equal numbers of chromosomes (Fig. 1), secondly that the polar bodies extruded by egg cells were also cells (Van Beneden 1883; Fig. 1) and, thirdly, that parthenogenetic eggs (which developed in the absence of fertilization) extruded only one polar body compared to the normally observed two in non-parthenogenetic sexually reproducing organisms (Weismann 1887). From this latter observation Weismann (1887) in a brilliant piece of deductive reasoning argued that “sexual reproduction can (only) proceed by a reduction in the number of ancestral germ-plasms, a reduction that is repeated in every generation*.* This *must* be so: the only question is, how and when does this supposed reduction take place”. In other words, he predicted the occurrence of meiosis.

**Fig. 2:**
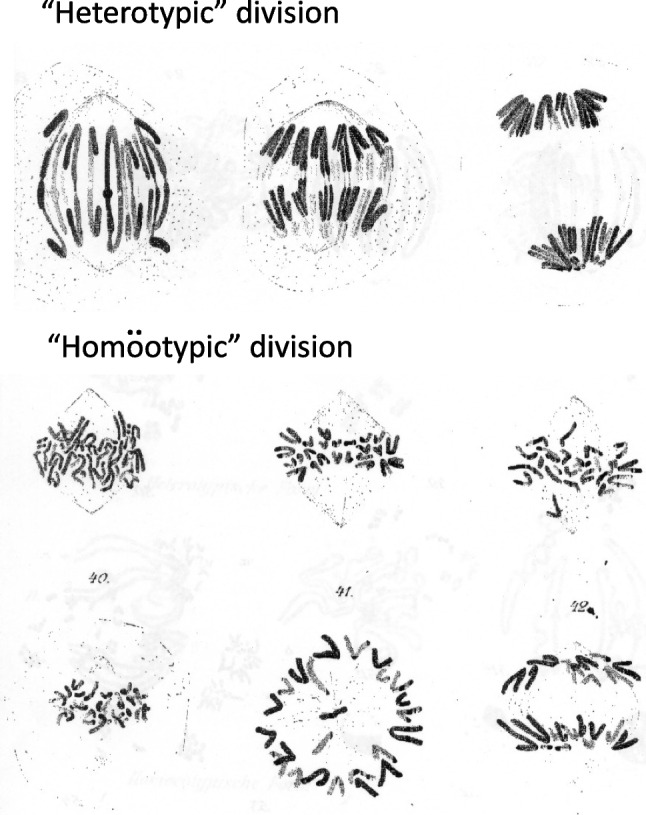
Discovery and demonstration by Flemming (1887) that the two spermatogenic divisions in the amphibian *Salamandra maculate* were fundamentally different, referring to them as “heterotypic” and “homöotypic” – now known as meiosis I and meiosis II

In parallel with Weismann’s deliberations, in the late nineteenth century there were numerous equally important and overlapping developments in nuclear cytology, aided by improvements in microscope lens technology and the development of better nuclear/chromosomal stains. This was a period of intense cytological activity into both “reduction” and fertilization involving many prominent investigators including Hertwig, Fol, Van Beneden, Boveri, Flemming, Henking, Strasburger and many others (see excellent review by Whitehouse 1973). Gradually, some sense emerged from disorder. Notably, in 1887, Flemming published his landmark paper “Neue Beiträge zur Kentniss der Zelle” (New contributions to the knowledge of the cell) in which he distinguished between the two spermatogenic nuclear divisions in the amphibian *Salamandra maculata.* Based on his detailed knowledge of somatic (mitotic) cell division he proposed that the two spermatogenic divisions were fundamentally different, referring to them as “heterotypic” and “homöotypic” – which we now refer to as meiosis I and meiosis II (Fig. 2). In the succeeding years, following these discoveries, the main mechanical framework of meiosis was largely understood although many details remained to be clarified. Prominently, there was the realization that homologous chromosomes (now often referred to as "homologs") are separated (disjoined) into daughter nuclei during meiosis I whereas meiosis II involves the separation of the chromatids of the disjoined chromosomes.

## Chiasmata and crossovers

As someone once remarked “Before you can divorce, you must first be married”. So it is, in the vast majority of cases, with chromosomes. In mitosis (and meiosis II), sister chromatids must be connected to know that they must segregate to opposite poles. In meiosis, analogously, orderly segregation of homologs at anaphase of meiosis I is dependent on their pairing plus mechanisms to transiently maintain their physical association while they achieve co-orientation on the metaphase I spindle. In both cases, connectedness is ensured by complex regulatory events in which "biorientation" (of sisters or homologs) sets up tension on centromere/kinetochore complexes which, when present for all chromosome pairs, licenses onset of anaphase. The special challenge for meiosis, then, is how to connect homologs.

**Fig. 3:**
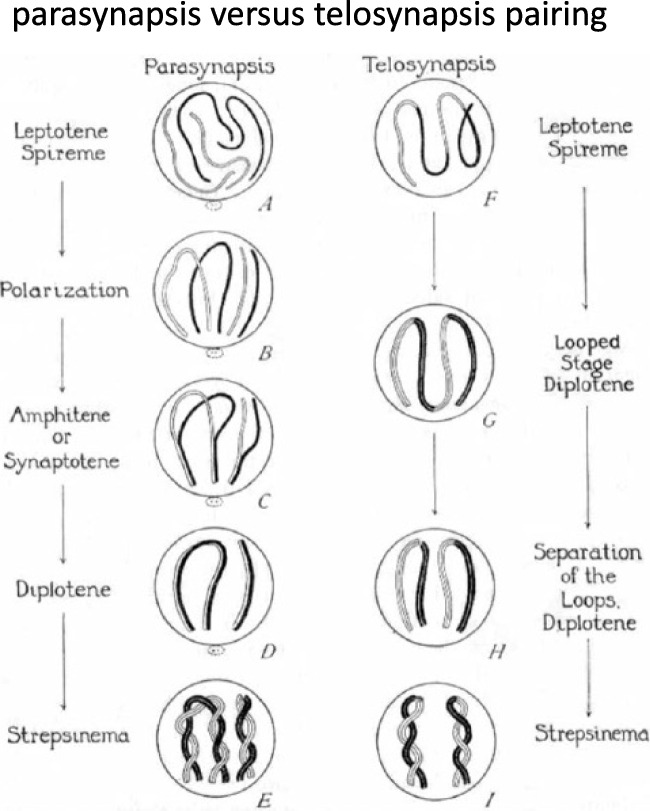
Long standing question: how do homologous chromosome associate during pairing? Early dispute (illustrated by Wilson 1925; Fig. 274) as to whether homologous chromosomes associated side by side (parasynapsis, left) or end to end (telosynapsis, right). His legend says: "Diagram showing the relation between “parasynapsis” and "telosynapsis" by loop-formation. In the parasynaptic series, one pair of loops and one pair of rods are shown. The final stages are much alike in effect (parasynaptic association of the synaptic mates), but the early stages are widely different"

**Fig. 4:**
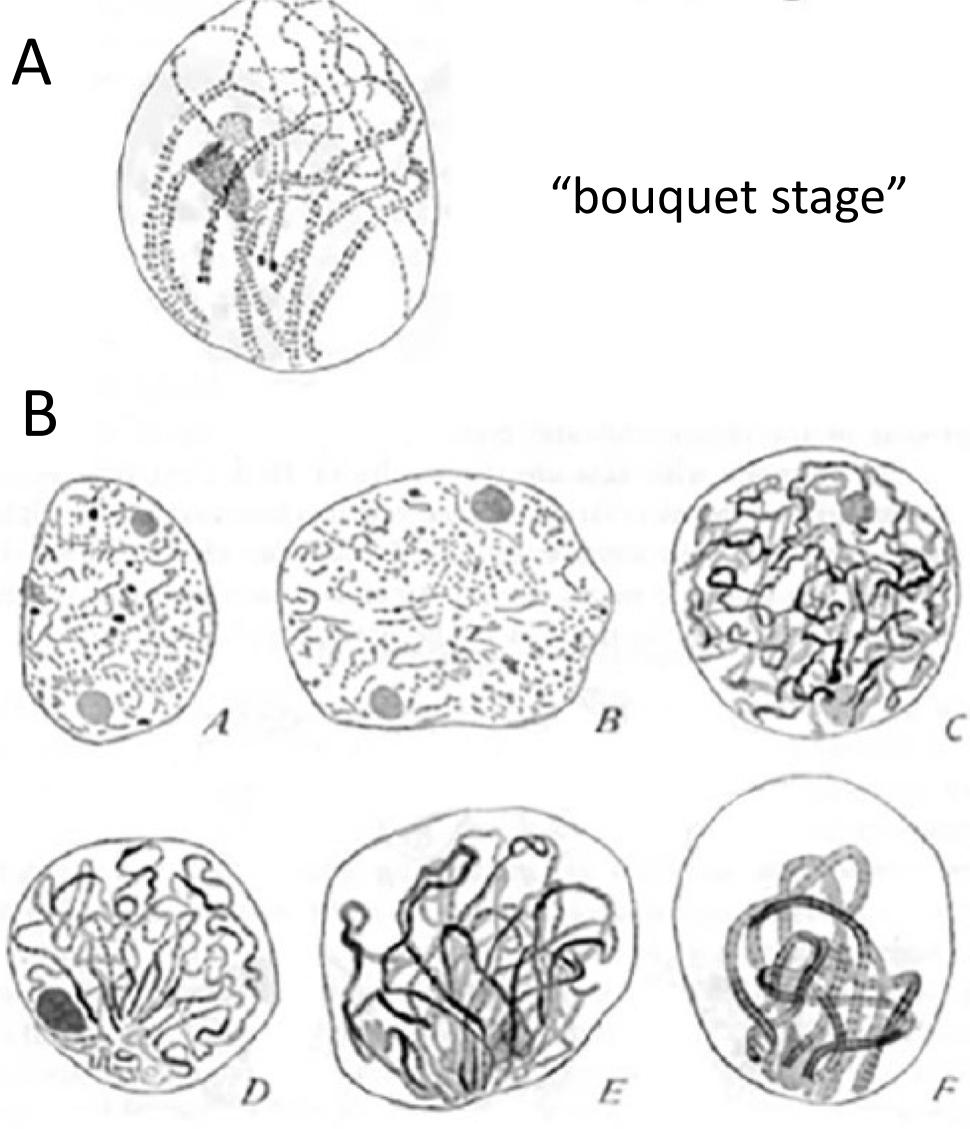
Detailed cytological observations on meiotic prophase I demonstrated side-by-side synapsis of homologs. **A** Grasshopper (from Wenrich 1916). Note that the clear drawing shows a zygotene stage where homolog ends are paired/synapsed (bottom) in a bouquet configuration while the middle part of the homologs are not yet coaligned (top). **B** Drawing of Triclade *Dendrocoelum* meiotic prophase I by Gelei (1921). (**A-C)** progression of axis formation and (**D-F**) of tight bouquet formation from late leptotene (left) to pachytene (right)

It was well-appreciated that during the prolonged prophase that precedes meiosis I chromosome segregation, homologs associate in pairs into "bivalents", presumptively as a precondition for their eventual disjunction. However, a dispute arose to whether homologous chromosomes associated side by side (parasynapsis) or end to end (telosynapsis) (Fig. 3A). This dispute was only resolved in favor of the former proposition by detailed cytological observations on meiotic prophase I in the spermatocytes of grasshoppers (Wenrich 1916) and flatworms (Gelei 1921) (Fig. 4A and 4B), thereby confirming the much earlier observations of von Winiwarter (1900) on the progressive development of oocytes in neonatal rabbits (see details in Whitehouse 1973).

**Fig. 5:**
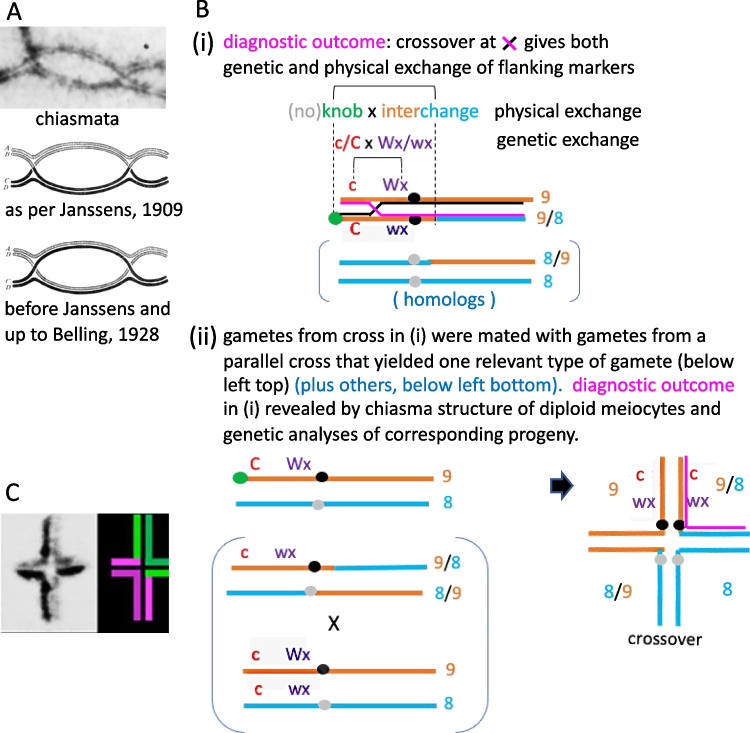
Chiasmata and physical exchange of non-sister chromatids at crossover sites. **A** top: Two chiasmata at diplotene from *Locusta migratoria* (G. Jones): arrows point to the two chiasmata sites. Middle: chiasma structure as points of physical exchange between the (non-sister) chromatids of homologous (black and white) chromosomes as interpreted by Janssens (1909). Bottom: alternatively, before Janssens and up to Belling (1928), chiasmata were represented as points when homologous chromosomes came together at diplotene but without physical exchange. **B** Creighton and McClintock (1931) showed that genetical crossovers coincide with physical exchange of chromosome segments by examining the output of meiosis in a maize line heterozygous for two genetic markers (C/c and Wx/wx) and flanking physical markers (a knob and a reciprocal translocation breakpoint). Panel (i) shows the heterozygous configuration at pachytene with a single crossover between the genetic markers which should be accompanied by physical exchange of the flanking physical markers. To reveal this outcome, gametes from the cross in (i) were mated with gametes from the line in (ii). Panel (iii, right) shows the resulting diagnostic chromosome configuration, defined by combined genetic and cytological analysis (these drawings and further explication in Coe and Kass 2005).** C** Proof that chiasmata coincide precisely with points of physical crossover exchanges, and that chiasmata do not migrate from their initial sites to more distal locations, was provided by differential BrdU labelling of sister chromatids in *Locusta migratoria* (adapted from Jones and Tease 1981)

Early investigators such as Rückert (1892) further observed that during meiosis I the chromosomes, which are now much shorter and fatter, appeared to be connected only locally by one or a few cross-shaped structures now termed chiasmata (Fig. 5A top). Later Janssens (1909) expanded on these observations and, in his “chiasmatype theory” proposed, with considerable insight, that these represent points of physical exchange between the (non-sister) chromatids of homologous chromosomes (Fig. 5A middle). A contrary interpretation, favored by many cytologists because it preserved the integrity of chromosomes, was that these chiasmata represented association of sister and non-sister chromatids, without physical exchange (e.g. Belling 1928; reviewed in Koszul et al. 2012; Fig. 5A bottom). Finally, after much debate and various cytological tests a consensus was arrived at that crossing-over takes place between two chromatids of different chromosomes at the chiasma before it is formed. A commentary of Janssens' landmark paper, prepared for its 100th anniversary, places his discovery in the context of experimental analyses of the time, with an accompanying English translation (Koszul et al. 2012).

Coincidentally, three parallel experiments in maize, *Drosophila melanogaster* and *Neurospora crassa* (Creighton and McClintock 1931; 1935; Stern 1931; Lindegren 1933) established that genetical crossovers coincide with physical exchange of chromosome segments (e.g. Figure 5B for Creighton & McClintock 1931). However, this had no direct bearing on the interpretation of cytological chiasmata. Naturally, a corollary of the now established interpretation of Janssens' chiasmatype hypothesis is that, in chiasmate meiosis, sister chromatids are intimately and strongly connected by a cohesion mechanism, and an associated process operates to undo this cohesion along sister chromatids arms at the onset of anaphase I. Also, cohesion at centromeres is differentially retained and then used to ensure regular sister chromatid segregation at meiosis II (review in Kudo et al. 2006).

Despite these important advances there was a residual concern that cytologically observed chiasmata from diplotene onwards may not coincide with original crossover positions. This arose because in many species, chiasmata are located towards the ends of bivalents observed at metaphase I. This led C. D. Darlington (1931) to propose that chiasmata could move, or slip, towards the bivalent ends, a process he called “terminalisation”. He embraced this idea because it seemed to support the parasynapsis, as opposed to telosynapsis, interpretation of chromosome pairing (above). In reality, it is now understood that the sub-terminal location of chiasmata in many species is a consequence of the distal localization of crossovers and the apparent movement of chiasmata reflects the fact that in most cases it is not possible to distinguish between chiasmata and relational twists of homologous chromosomes. Finally, observation in Orthopteran insects where the four chromatids are clearly visible until metaphase I and therefore chiasmata can be unequivocally identified (Fig. 5A), plus differential BrdU labelling of meiotic chromatids in *Locusta migratoria,* conclusively proved that chiasmata coincide precisely with points of physical crossover exchanges (Tease and Jones 1978; Jones and Tease 1980; Fig. 5C) and that chiasmata do not migrate from their initial sites to more distal locations (Jones and Tease 1980).

## Crossover patterning and interference

The distribution of crossovers/chiasmata is known to be decidedly non-random. For example, even in situations where chiasmata are not strongly localized distally or proximally, quantitative studies reveal peaks and troughs of chiasma frequencies along meiotic bivalents (e.g. Jones 1986). This tendency for crossovers (COs) to be spaced out along chromosomes dates back to early genetic studies in *D. melanogaster* (Sturtevant 1913; 1915; Muller 1916). These studies, made in the context of experiments designed to show that genes occurred in a linear order, revealed that double COs between nearby markers are less frequent than expected from the individual frequencies of single COs (Sturtevant 1915; Muller 1916). This condition, referred to as "crossover interference" by Muller (1916), implied that COs do not occur independently of each other. It is as if a CO at one position "interferes" with occurrence of another CO nearby. Genetic analyses also showed that the strength of interference decreases with increasing distance between the two COs positions (review in Jones 1987; Zhang et al. 2014a, b). The overall effect is that CO tend to be more or less evenly-spaced along bivalents. CO interference is, therefore, a major determinant for the number and positions of COs along each pair of homologous chromosomes in almost all studied organism, with the known exceptions of fission yeast and *Aspergillus nidulans* (review in Jones and Franklin 2006; Berchowitz and Copenhaver 2010; von Diezmann and Rog 2021; Zickler and Kleckner 2023). The nature of interference could be described quantitatively from extensive genetic data by Muller (1916) who subsequently introduced for this purpose the classical approach of Coefficient of Coincidence curves, which plot the ratio of observed to expected double COs (the "CoC") for all pairs of intervals as a function of inter-interval distance.

The effect of interference is also apparent in the physical distancing of adjacent chiasmata along meiotic bivalents (e.g. in the plant *Vicia faba*, Haldane 1931). A more rigorously quantitative approach arose from exploiting the unusually clear chiasmate diplotene bivalents of the Orthopteran *Chorthippus brunneus*. By a detailed analysis of distribution along a large number of bivalents, Laurie and Jones (1981) showed that several features of chiasma distribution are strongly indicative of the operation of interference; e.g. they found evidence of a minimum interchiasma distance equal to 30% of the physical length of the long arm, irrespective of the locations of chiasmata on the arm, which implies the presence of complete interference in the immediate vicinity of each CO. This and other studies (e.g. by plotting inter-chiasma distances in histogram forms, Jones 1986) found that chiasma interference, measured as the coefficient of coincidence, was complete over 25–30% of the bivalent arm, and then diminished gradually over the next 30% of the arm. These studies, and a recent genetical analysis of CO interference across the entire human genome have also established the important fact that, contrary to early dogma, interference acts across centromeres (e.g. Harte 1956; Colombo and Jones 1997; Broman and Weber 2000).

In modern studies, interference is often (also) analyzed cytologically at pachytene by monitoring the positions of CO-correlated fluorescent foci along synapsed homolog bivalents (e.g. Zhang et al. 2014c). Such studies reveal that, mechanistically, the distance over which the interference effect manifests itself is a function of physical distance along the chromosomes, in microns (Drouaud et al. 2007; Zhang et al. 2014c), rather than the "genetic distance" (cM) used in early Drosophila studies or "genomic distance" (bps of DNA), now also available from DNA sequencing studies (e.g. Martini et al. 2006; Petkov et al. 2007). Importantly, the existence of CO interference implies the presence of communication along the chromosomes or, as sometimes now stated, the positions of COs are determined by "one-dimensional spatial patterning" (see discussion in Zickler and Kleckner 2023).

## The obligatory crossover

**Fig. 6:**
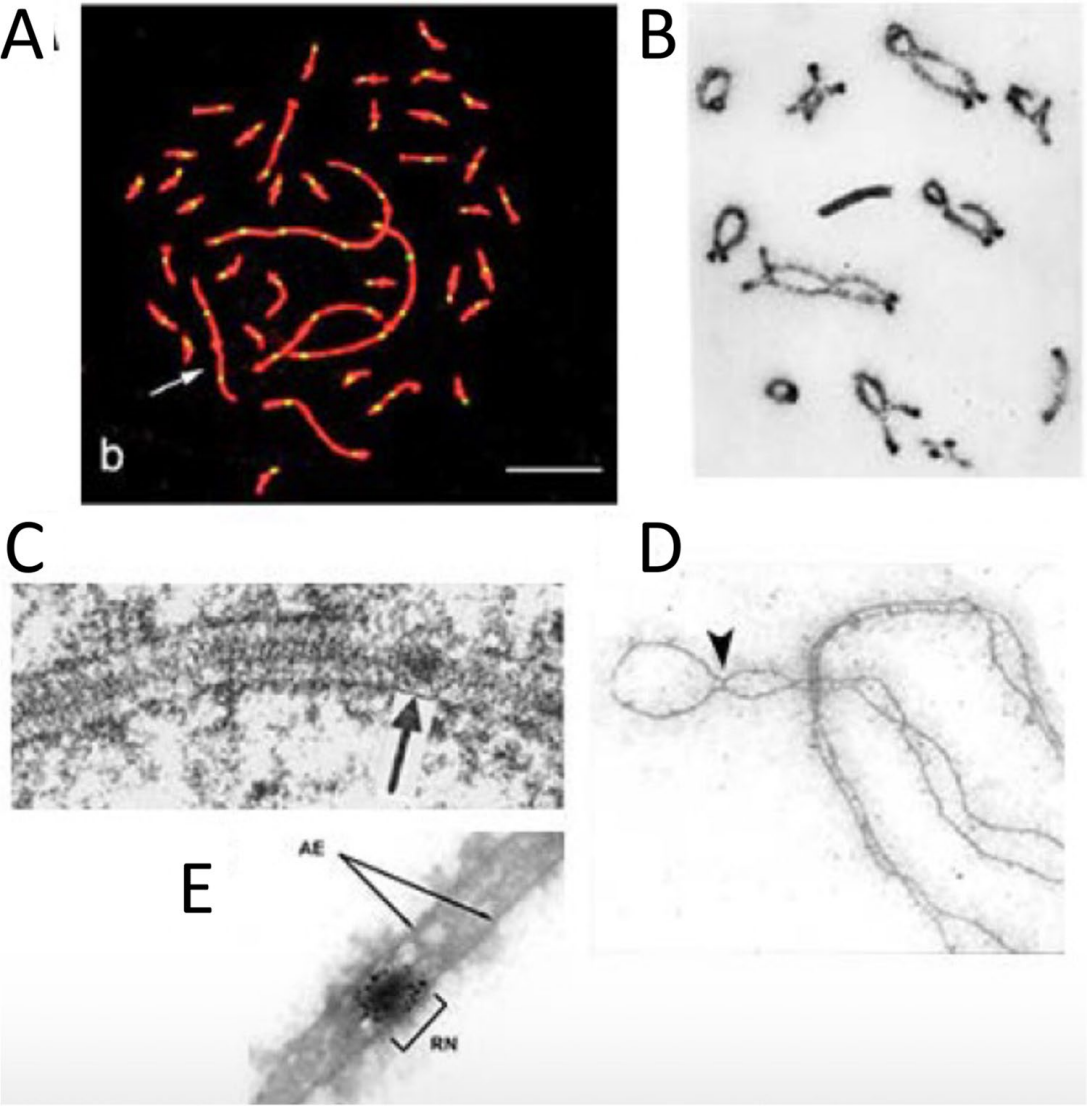
Synaptonemal complex and recombination nodules.** A** MLH1 foci ( white) along pachytene chromosomes (red) in zebra finch (*Taeniopygia guttata)*, 2n = 80 comprising 14 macro-chromosomes and 64 micro-chromosomes (Calderon and Pigozzi, 2006).** B** Diplotene chiasmata in desert locust *Schistocerca gregaria* with different sizes of chromosomes (Jones and Franklin 2006).** C** EM image of SC and EM-defined SC-associated nodule (arrow) that correspond to crossover recombination complexes, in *D. melanogaster* female. Note that the nodule does not penetrate the SC central region (Carpenter 2003). **D** EM spread of coaligned homolog axes linked by bridges (arrowhead) in spread preparations of *Allium fistulosum* (Albini and Jones 1987). **E** EM-defined SC-associated nodule (RN) is confirmed as a crossover site by immunogold colocalization of crossover factor Mlh1 (Lhuissier et al. 2007)

An even more striking manifestation of the non-randomness of COs/chiasmata is the nearly absolute rule that homologous-chromosome pairs almost always have at least one CO (e.g. no chiasmata, that is univalence, in only 4 among 25,120 pairs in *C. brunneus* spermatocytes, Jones 1987; review in Jones and Franklin 2006). This is true regardless of chromosome size (e.g. in the Zebra finch *Taeniopygia guttata* with 14 macro chromosomes and 64 micro chromosomes or the Japanese quail with 7 macro chromosomes and 31 micro chromosomes, each with at least one Mlh1 focus (Fig. 6A; Calderón and Pigozzi 2006). This feature is often referred to as the "obligatory crossover" because it reflects the fact that at least one CO per bivalent is required to satisfy the mechanical requirement for homolog connectedness at the first meiotic division. Thus, from the point of view of the meiotic process per se, this is probably the most important feature of CO non-randomness. In the standard meiotic program, occurrence of the obligatory CO is a specific, programmed effect. This is emphasized by the fact that, in the vast majority of organisms, the total number of COs per bivalent is very small, often two or a handful, and sometimes one and only one (review in Mercier et al. 2015); but the obligatory CO rule is nonetheless observed.

The lengths to which organisms go to ensure the obligatory CO is further manifested in the fact that it is observed: (i) irrespective of the total number of chromosomes (from two in the worm *Parascaris univalens* and in the Australian daisy to over 1400 in some adders-tongue ferns like *Ophioglossum reticulatum*); (ii) in species having a wide range in chromosome sizes like the grasshoppers *Schistocerca gregaria* (Fig. 6B) and *C. brunneus* (Jones 1986); (iii) in situations where the opportunity for COs is spatially restricted, e.g. when the chromosome is very short (e.g. Murakami et al. 2021; Calderón and Pigozzi 2006), when a majority of COs arise in less than a quarter of the genome due to the presence of large blocks of heterochromatin (e.g. Mercier et al. 2015) and for the XY sex chromosomes of mouse and human, where the available homologous region ("PAR") is tiny compared to total chromosome lengths (Kauppi et al. 2012). However, in few organisms, notably fission yeast and *Aspergilus nidulans* (which, notably, also lack CO interference; above), occurrence of at least one CO per bivalent is achieved by having a sufficiently large number of COs (~ 10–20 per bivalent in both cases) that, by chance, the probability of zero events is extremely low.

It is now known that occurrence of at least one (obligatory) CO depends on two factors. First, at least one pre-CO recombination interaction must be designated to mature along a CO-specific pathway. This effect is likely to be an intrinsic consequence of the same CO patterning process that gives rise to CO interference (Zhang et al. 2014a, c; Zickler and Kleckner 2023). Second, the CO maturation process must occur efficiently, an effect sometimes referred to as "CO assurance" to indicate that additional events are required after "CO designation" to "assure" that a CO product actually occurs (Shinohara et al. 2008).

### Ultrastructure analysis: the synaptonemal complex and recombination nodules.

#### The synaptonemal complex

Ultrastructural electron microscopic (EM) studies of meiosis commenced in the 1950s with remarkable and still not fully understood findings. First, Moses (1956) and Fawcett (1956) revealed the presence of a tripartite core-like structures called synaptonemal complexes (SCs), that run along the entire lengths of the homologs. SCs have further been found in most plants, animals, fungi, algae and protozoans that reproduce sexually (with the known exceptions of fission yeast, *A. nidulans, D. melanogaster* male and the protist *Tetrahymena thermophila*, review in Loidl 2021). The SC forms by linking the structural axes of two homologs by a close-packed array of transverse filaments which emanate towards one another from the two axes and with additional specific proteins located at the points where they converge (review in Page and Hawley 2004; Fraune et al. 2016; Zwettler et al. 2020). By EM, these features comprise the SC "central region" and its component "central element" which defines the midline of the central region along its length (Fig. 6C).

The SC was shown to normally form between homolog axes, as illustrated for example in reciprocal translocations, where chromosome segments are exchanged between two non-homologous chromosomes (review in von Wettstein et al. 1984). However, when no partner is available, SCs can assemble between nonhomologous axes in which the two axes are close together (e.g. in foldback chromosomes), between non-homologous chromosomes in haploid meiosis or between the three homologous chromosomes in triploid strains (examples in Rasmussen 1977a, b; Rasmussen et al. 1981; von Wettstein et al. 1984; Zickler and Kleckner 1999). Also, as observed in many species, SC components have a tendency to self-assemble outside of chromosomes, e.g. when the normal assembly process is defective, leading to stacks of SCs called polycomplexes (review in Hughes and Hawley 2020). Recent studies have now revealed that SC central region components are highly dynamic, suggesting that the structure meets the definition of a liquid crystal (Rog et al. 2017; Zhang et al. 2020).

Interestingly, the SC can also play a direct role during meiosis I segregation: in achiasmate meioses (like in silk-worm females), a “modified SC” remains between homologs and provides thus the connection required for their proper segregation at Anaphase I (Rasmussen 1977a, b, review in Zickler and Kleckner 1998). It was, however, shown recently that the so-called modified SC, is in fact formed by the fusion of two SC lateral components HOP1 and SYCP2 with a thick HOP1 band sandwiched by two layers of SYCP2. The authors propose to rename this structure “bivalent bridge” (Xiang et al. 2024).

EM analysis of 3D reconstructions and spread preparations of mammals, plants and fungi revealed that before SC formation, the axes of homologs are first coaligned at a distance of ~ 400 nm (e.g. Albini and Jones 1987; Anderson and Stack 1988; Zickler 1977; 2006). Such images revealed that the fundamental task of homolog recognition and juxtaposition precedes SC formation, with genetic studies further showing that both processes are dependent on recombination (e.g. Storlazzi et al. 2003; Dubois et al. 2019). Furthermore, coaligned axes are sometimes linked by EM-visible bridges that often exhibit nodular structure (Fig. 6D; Albini and Jones 1987; Moens et al. 2002; Anderson and Stack 1988; Anderson et al. 2001); and sometimes nodules also occur in association with individual homolog axes which are not visibly linked to any partner (Albini and Jones 1987; Anderson et al. 2001; Moens et al. 2002; 2007). Such images suggested that recombination complexes are associated with chromosome structures (axes/SCs) from very early (presynaptic) stages onward and that SC nucleation occurs at sites of recombination interactions via bridge structures (Dubois et al. 2019).

These suggestions were directly validated by: (i) fluorescence imaging of axes, bridges and recombination complexes both before, and at, the coalignment stage (e.g. Oliver-Bonet et al. 2007; Moens et al. 2007; Storlazzi et al. 2010; Dubois et al. 2019); (ii) EM documentation of SC nucleation at sites of recombination nodules (Zhang et al. 2014a); (iii) identification and molecular analysis of SC-nucleating bridges, which were shown to be “mini-axes” containing cohesin plus recombination components (Dubois et al. 2019) and (iv) that bridges mediate transfer of recombination complexes from on-axis to between axis/on-SC localization (Dubois et al. 2019; review in Zickler and Kleckner 2023).

#### Recombination nodules

Ultrastructural analyses also revealed nodule-like structures associated with SCs (Fig. 6C). In a groundbreaking study of *D. melanogaster* oogenesis Carpenter (1975) argued that EM observed nodules at pachytene represent the sites of genetical COs because they exhibited the same pattern of obligation and interference that had been detected by genetic studies and called them “recombination nodules”. Other evidence for a correspondence between COs and SC-associated nodules was provided by the observation that in the plant *Allium fistulosum* nodules were proximally localized, in agreement with the distribution of chiasmata observed at later stages (Albini and Jones 1988). Many subsequent investigations identified similar nodules in several plant, animal and fungal species and showed that: (i) every SC had at least one nodule, (ii) that they corresponded to the number of chiasmata, (iii) that they displayed interference and, finally, that they were maintained at chiasma sites (with remnants of SC pieces) up to diakinesis (reviewed in von Wettstein et al. 1984; Zickler and Kleckner 1998; 1999). Also, EM analysis of recombination-defective mutants showed that the number and distribution of nodules parallels the number and distribution of genetically observed COs (Carpenter 1979; Zickler et al. 1992). Subsequent fluorescence imaging studies confirmed this correspondence to CO recombination machineries: recombination-associated proteins (notably MLH1/3, implicated directly in late stages of CO formation, and the E3 ligase Hei10) co-localize with such nodules at both EM and LM levels (e.g. Marcon and Moens 2003; Anderson et al. 2014).

These EM-visualized structures that correlated with COs later became known as "late recombination nodules" because observations made on earlier prophase I stages described another type of nodules, often smaller, much more numerous and already seen at the "zygotene" stage (Carpenter 1987; von Wettstein et al. 1984; Albini and Jones 1987; Bojko 1989; Anderson and Stack 2005). These were first termed "zygotene nodules" and, later, early recombination nodules. However, in some organisms (e.g. in the fungus *Sordaria macrospora* and tomato), nodules that correlate with COs and are morphologically distinct from the other, more numerous nodules, are already present at zygotene (Lhuissier et al. 2007; Zhang et al. 2014a). Immunogold staining in EM and recent light-fluorescence imaging studies showed that those early nodules correspond to the localization of recombination proteins like Rad51, RPA, Mer3 and Msh4/5, required for early stages of recombination (e.g. Moens et al. 2002; de Boer et al. 2006; Oliver-Bonet et al. 2007; Storlazzi et al. 2010; Yokoo et al. 2012) while late nodules correspond specifically to proteins involved in formation of crossovers (e.g. Mlh1, Fig. 6E; Lhuissier et al. 2007; Anderson et al. 2014).

It is now clear from many studies of meiosis that a large number of recombination interactions are initiated very early, among which only a few ultimately mature into COs. That is: "many are called but few are chosen" (to be COs). This relationship emerged originally from genetic studies of recombination in fungi which identified and analyzed non-Mendelian marker segregation, which is diagnostic of DNA/DNA interactions for recombination, as well as CO (Lindegren 1933; Zickler 1934; reviewed by Whitehouse 1982). Non-Mendelian segregations were found to be much more frequent than COs. Moreover, non-Mendelian segregation of a particular allele could be accompanied, or not, by crossing-over between markers at flanking positions, with the CO outcome usually being the rarer of the two (review in Whitehouse 1982). Put more specifically: many DNA/DNA interactions occur, among which a small subset is matured as COs (with accompanying interference and obligation) while the remainder are matured without crossing-over, i.e. as "noncrossovers" (review in Hunter 2015). The so-called "early nodules" may correspond to total recombination complexes and/or only to those that will not become COs and thus have been left behind, according to the situation. Presciently, Carpenter proposed that early nodules corresponded to noncrossovers and that the excess of such interactions were more abundant than COs because they were involved in homolog pairing (Carpenter 1987).

Further analysis of EM data revealed that, in Sordaria, SC nucleation sites are evenly spaced (in accord with observation from *Allium* images Albini and Jones 1987) and confirmed the implication that the classical interference process, which gives evenly-spaced COs along the chromosomes, actually acts more broadly to give a larger number of evenly-spaced SC nucleation sites, a subset of which are (interfering) CO sites (Zhang et al. 2014a). This and other findings point to the occurrence of a designation/interference process that operates at the structural level, with classical CO interference resulting from coupling of recombination complex status to that process (see discussion in Zickler and Kleckner 2023).

### The bouquet and interlockings: two sides of the pairing coin

Early cytological studies identified two interesting chromosome configurations that occur contemporaneously during the period when homologs are undergoing pairing and synapsis. One such configuration is the "bouquet", in which all chromosomal telomeres are tightly clustered in a small region of the nuclear envelope and, in classical images, homologs are not obviously paired. First described and named by Eisen (1900) in salamander meiosis and further described in detail by Gelei (1921; Fig. 4B), this configuration has since been found in most studied organisms (review in von Wettstein et al. 1984; Scherthan 2001; Zickler 2006; Zickler and Kleckner 1998; 2016). A second interesting configuration is whole chromosome "interlocking", in which one chromosome, or a pair of homologs, is trapped within an open, unpaired region of another chromosome pair (examples in von Wettstein et al. 1984; Rasmussen 1986). Interlocked configurations emerge during the pairing period but are then mostly actively resolved by the end of prophase, with only regular bivalents seen by the end of pachytene (e.g. Storlazzi et al. 2010).

The bouquet configuration was originally proposed to occur prior and prerequisite to pairing of homologous chromosomes as a way of topologically simplifying the homology search process (e.g. Gelei 1921; Scherthan 2001). Recent studies reveal that the bouquet configuration is the result of active telomere-led movements driven by the cytoskeleton and that it can be quite transient (review in Link and Jantsch 2019). Early studies also identified global whole-nucleus rotations (Parvinen and Soderstrom 1976), now also understood to be mediated by cytoskeletal forces. Diverse lines of evidence suggest that while the bouquet configuration and or cytoskeleton-mediated movements may promote juxtaposition of homologs, chromosome motions also appear to provide a stringency factor which has the potential to eliminate both unwanted pairwise associations and whole chromosome interlockings (e.g. review in Link and Jantsch 2019; Klutstein and Cooper 2014; Zickler and Kleckner 2016 and references therein).

Importantly, also, elimination of interlocks requires not only regularization of whole chromosome topological relationships but resolution of constraining DNA interactions. This is revealed by the finding, in Sordaria, that interlock resolution requires Mlh1 (Storlazzi et al. 2010). Mlh1 is a subunit of a structure-specific nuclease that not only plays a central role in crossover formation (Hunter 2015) but also disrupts developing CO-fated recombination complexes in the presence of base-pair mismatches (Hunter and Borts 1997). It has also been suggested that DNA topoisomerase II activity may be involved in interlock resolution (von Wettstein et al. 1984; Martinez-Garcia et al. 2018).

### Studies of meiotic mutants and chromosome aberrations

It was quickly understood that dissection of the mechanisms underlying the transitions through the complex events of the meiotic process required the use of mutants (e.g. Sandler et al. 1968; Baker and Hall 1976). Therefore, through different strategies (e.g. sensitivity to radiation or chemical mutagens, abnormal segregation, reduced fertility, research in natural populations, etc.), several laboratories began a systematic search and study of mutations that affected one or more of the meiotic processes. A considerable number of mutants that modify the normal pattern of meiosis have been isolated in *D. melanogaster*, fungi (budding and fission yeasts, *N. crassa*, *Podospora anserina*, *Aspergillus nidulans*, *Ustilago maydis*) and plants (maize, rice, wheat) (review and corresponding references in Baker et al. 1976). Interestingly, most of the isolated mutants showed abnormalities during the first meiotic division (likely because perturbation of its complex program confers extreme, easily-detectable phenotypes) and few were defective for events occurring during the second division (Baker et al. 1976).

However, the functions of the corresponding gene products were only mostly elucidated when molecular-biology tools were available. One illustration: among the several meiotic mutants known for *D. melanogaster* (review in Sandler et al. 1968; Baker et al. 1976), the *crossover suppressor in chromosome 3 of Gowen c(3)G* mutant, which was one of the first *bona fida* described meiotic mutant (Gowen and Gowen 1922), was shown 79 years later to correspond to a gene encoding the SC transverse filament protein, ortholog of the budding-yeast Zip1 protein (Page and Hawley 2001). Similarly, one of the first *bona fide* meiotic mutants of budding yeast, *SPO11* (Klapholz et al. 1985), turned out to encode the topoisomerase-related transesterase responsible for recombination-initiating DSBs (Keeney et al. 1997). Indeed, much of the early progress in the identification of genes coding for recombination and SC proteins was produced by studies in budding yeast, in which isolation of mutants could be coupled with biochemical, physical and genomic analyses. Other prominent early examples include identification of meiotic and eukaryotic RecA homologs (Bishop et al. 1992) and the first-identified SC transverse-filament protein (Sym et al. 1993).

Another successful approach for isolation of new genes involved in the meiotic process, is the screen for suppressors which allow either the reversal of the original mutation (back to wild type, or close to it), or, more often, correspond to mutation of a different gene that suppresses the effect of the original mutation. Those mutants have often the advantage of a minor fertility defect, which might have escaped in a forward screen. For example, chemical mutation of *A. thaliana* seeds from *zip4 or msh5* mutants with low fertility, allowed the recovery of four factors including two helicases FANCM and RECQ4, the AAA-ATPase FIGL1, and the FIGETIN-LIKE-1 INTERACTING PROTEIN (FLIP) that increase the frequency of class II COs (Mus81 pathway), also conserved in rice, pea and tomato (Crismani et al. 2013; Mieulet et al. 2018). Also, suppressors of the meiotic arrest at metaphase I of a non-null Sordaria mutant of *SPO76/PDS5* allowed the recovery of 32 mutants belonging to seven genes called *ASY1* to *ASY7* because of their pairing and synapsis defects (Huynh et al. 1986). Their ensuing sequencing showed that this approach allowed the isolation (and study) of the almost complete cohort of genes (*SPO11*, *SKI8*, *Topoisomerase VIB-like, MER2, REC114*, *SAE2* and *MRE11*) required for DSB formation (e.g. Tessé et al. 2003; 2017), now known from budding yeast studies to form a molecular condensate (Claeys Bouuaert et al. 2021).

Systematic searches for mutants also advanced our understanding of how chromosome aberrations behave during meiosis (e.g. Perkins 1997 for filamentous fungi).

Three illustrations: (i) The classical genetic studies in *D. melanogaster* revealed that the frequency of COs was reduced between two homologous chromosomes if one homolog carried either an inversion or a translocation but that also, more intriguingly, the resultant reduction in exchange was accompanied by an increase in CO on other pairs of normal-sequence chromosomes, without abrogation of CO interference (Sturtevant et al. 1919; 192,1; Crown et al. 2018; review in Miller et al. 2020). This phenomenon, termed the "interchromosomal effect" has subsequently also been observed in several plants (references in Jones 1987; Termolino et al. 2019). Increased CO formation can result from arrest of prophase (Joyce and McKim 2011), perhaps because this allows a longer period for CO designation. (ii) Analysis of SC and recombination nodules in a heterozygous inversion of maize showed that SC forms in the inverted region, in association with the presence of a recombination nodule, thus in correspondence to underlying homology, pointing to the then-unexpected possibility that crossover sites nucleate SC formation (Maguire and Reiss 1996). (iii) EM analyses allowed to discover cases in which SC formation within a region harboring a deletion or an inversion is initially strictly homologous; this pattern is followed by a “correction phase” in which only continuous two-by-two associations are observed, as illustrated by “synaptic adjustment” in mouse and *N. crassa* inversion loops (Moses and Poorman 1981; Bojko 1989). A different type of "two-phase synapsis" is observed in allohexaploid wheat, where SC initially forms among homeologous and homologous pairs of partners and then adjusts such that only homologous pairs are observed, both for CO interactions and for synapsis (review in von Wettstein et al. 1984). These dynamic patterns are likely facilitated by the fact that SC central-region components are constantly moving in and out of the structure (Rog et al. 2017).

Systematic analysis of mutants with affected fertility allowed also the discovery of other interesting processes. One example is meiotic silencing. Some organisms (e.g. the fungus *Neurospora crassa*) develop the ability to scan homology along their chromosomes at meiosis onset and silence any DNA segment lacking a homologous allelic partner by a process called meiotic silencing by unpaired DNA (MSUD; Shiu et al. 2001). The mechanism(s) by which unpaired DNA is detected remains unknown, but it does not depend on recombination and may involve direct DNA/DNA recognition between intact duplexes (Gladyshev and Kleckner 2017; Mazur and Gladyshev 2023; Rhoades et al. 2021). Additionally, among the factors required, several are RNAi proteins that target homologous mRNAs for silencing, RAD54 helicase and the meiotic-specific cohesin Rec8 (review in Hammond 2017; Rhoades et al. 2021).

### Budding yeast and the passage to the molecular biology era

In the 1980's, budding yeast emerged as a powerful system for combining cytological, biochemical, molecular and genetic approaches to analysis of meiosis. Of special importance, in this system, large populations of cells can be taken synchronously through the meiotic program and multiple events of interest assayed, as a function of time, in the same culture.

Introduction of molecular markers, combined with physical analysis of DNA events by Southern blotting of one- and two-dimensional gels resulted in the discovery that meiotic recombination initiates by programmed double-strand breaks (DSBs) (Cao et al. 1990) which, subsequently, were shown to occur in nucleosome-free regions (Wu and Lichten 1994). Also, time course analysis allowed to show that DSBs occur just after replication, in G2/early leptotene, whereas COs occurred at the SC stage (pachytene) (Padmore et al. 1991), also shown since to be true in mouse spermatocytes (Guillon et al. 2005). This latter finding nicely matched the fact that CO-correlated recombination nodules occur at pachytene (above) and excluded early models in which CO formation and accompanying CO interference occurred after pachytene, at the time that chiasmata appeared, as driven by post-prophase compaction (Darlington 1937). Concurrent analysis of DNA and chromosomal events in mutants also provided the first evidence that events at the two levels are not only coordinated in time but also are functionally coupled. Among very early findings, DSB formation and DSB resection were found to be required for formation of axial elements and of SC, respectively (Alani et al. 1990).

 Other early studies were targeted at determining the timing with which recombination bifurcated into CO and noncrossover pathways and, thus, the time of imposition of CO interference (Storlazzi et al. 1995). Noncrossover recombination is also completed during pachytene, but somewhat earlier than CO formation, thus accommodating the presence of early nodules on SCs but with precipitous disappearance at early/mid-pachytene (Storlazzi et al. 1995; Allers and Lichten 2001; Teresawa et al. 2007; Stack and Anderson 2002; Guillon et al. 2005). More importantly, in budding yeast, the distinction between the CO and noncossover fates was shown to be made very early in the recombination process, prior to stable strand exchange, at the time that SC formation is initiated (review in Bishop and Zickler 2004). Two-dimensional gel analysis identified two stable post-DSB DNA intermediates, single-end invasions (SEIs) and double Holliday junctions (dHJs), both of which were found to be specific to formation of COs (Schwacha and Kleckner 1995; Hunter and Kleckner 2001; Börner et al. 2004). Moreover, SEIs appear at the same time as SC nucleation and certain mutations coordinately block both SC and CO formation, without impeding formation of noncrossovers. These findings plus cytological studies (Fung et al. 2004) showed that CO interference is imposed very early in budding yeast, during late leptotene, and thus in accord with the situation elucidated by EM in Sordaria (Zhang et al. 2014a). The fact that dHJs give only COs also implies the existence of directional bias in resolution of the two junctions, for which an attractive biochemical explanation has recently been suggested (Kulkarni et al. 2020).

Early highlights from budding yeast studies also included: (i) the fact that a mitotic DSB repair function, the Rad50-Mer11-Xrs2 complex, mediates both the formation and resection of DSBs (review in Lam and Keeney 2014); (ii) identification of Spo11 as the meiosis-specific protein that mediates DSB formation and its identity as a transesterase (rather than a nuclease) (Keeney et al. 1997); and (iii) discovery of the meiosis specific molecule Dmc1 and, concurrently its general counterpart Rad51, as the first eukaryotic RecA homologs identified in any organism (Bishop et al. 1992; Shinohara et al. 1992). The identities of these molecules further implied that meiotic recombination has evolved from mitotic DSB repair, as suggested by early studies from Game et al. (1980) and Prakash et al. (1980) and confirmed and extended by many subsequent studies. The early paradigm of Dmc1 and Rad51 further illustrates the fact that, in a number of cases, meiotic and mitotic homologs collaborate, but with accompanying modulation of the mitotic activity. Dmc1 and Rad51 colocalize on both DSB ends where, however, they have different functions: Dmc1 is the primary executor of strand exchange while Rad51 is utilized to define partner choice, i.e. to ensure that a DSB undergoes strand exchange with a homolog partner chromatid rather than with its sister (review in Brown and Bishop 2014).

Budding yeast studies also identified the first gene encoding an SC protein, Zip1 (Sym and Roeder 1995), the first genes for meiotic axis components (Red1, Hop1; Hollingsworth et al. 1990; Rockmill and Roeder 1988) and a group of proteins which act in concert with Zip1 at the SEI/SC nucleation transition point, referred to as ZMM or SIC proteins (Börner et al. 2004). Fluorescent foci corresponding to these latter molecules (e.g. Zip3) were found to correlate specifically with sites of COs; and cytological analysis of these foci in a *zip1null* mutant (and, later, other SC-defective mutants) demonstrated that SC formation is not required for the obligatory CO or CO interference in this organism (Fung et al. 2004).

In organisms other than budding and fission yeasts, direct DNA analysis of recombination has not been possible (respectively, above and reviews in Cromie and Smith 2008; Tsubouchi et al. 2021; Arter and Keeney 2023 and references therein). However, the timing of recombination progression can be inferred from fluorescence visualization of recombination complexes characteristic of different stages. By such criteria, the timing described for budding yeast is likely essentially universally conserved. This is exactly true in organisms where SC forms after DSB formation, as is the majority case. In *C. elegans* and *D. melanogaster* female, SC forms prior to DSB formation but the program of DNA events seems nonetheless to be conserved (reviewed in McKim et al. 1998; Yu et al. 2016). Also, because the basic principles of chromosome behavior and recombination are relatively conserved among species, budding yeast orthologs represented a powerful tool to identify meiotic genes in other fungal species, plants, mammals, nematodes, flies, fish, algae and protozoa (Zickler and Kleckner 2023 and references in corresponding papers). They were identified either by sequence homology of their corresponding genes, or by complementation of mutants identified before the molecular era (e.g. *D. melanogaster*, fission yeast, *S. macrospora*, above).

### Meiotic recombination—buffering against challenges to robust pairing and/or (obligatory) crossover formation

Recombination is important for three aspects of meiosis. First, DSBs mediate coalignment in most organisms, which will be compromised if the number of DSBs is reduced below some critical level (review in Zickler and Kleckner 2023). Second, CO recombination is most fundamentally important for meiosis to generate the first (the obligatory) CO as required for homolog disjunction (above). Regardless of mechanism, a CO will arise because a DSB-mediated "precursor" interaction undergoes CO designation. Thus, occurrence of the obligatory CO will require: (i) a sufficient number of DSBs that (ii) at least one resulting precursor will (iii) always be sufficiently sensitive to the available level of CO designation activity to undergo such designation. This progression can fail if there are not enough DSBs to provide the requisite sensitive precursor; if DSBs occur normally but precursors do not form due to a defect in pairing; if precursor sensitivities to crossover designation are reduced; or if the strength of that designation process is diminished. Third, formation of additional COs, beyond the obligatory one, may assist in promoting regular alignment of homologs on the meiosis I spindle (as well as having roles for evolution). The meiotic process includes diverse effects which buffer these three important outcomes against the possibility of inefficiency or disruption.

#### 1. When there is a paucity of DSBs

In this condition, homolog coalignment may be defective; the probability of the obligatory CO will be decreased as are overall total CO levels. This condition arises, and is accommodated (or not) in several situations.

1. Large amounts of heterochromatin. In some organisms (notably higher plants), a large fraction of the genome is organized as heterochromatin, in centromeres, telomeres and interstitial regions. Since DSBs are reduced, but not absent, in such regions, evolution has apparently provided conditions that can compensate for this deficit (Kuo et al. 2021).
2. Limited homology between sex chromosomes. In male mouse (also true in human), CO is restricted to the small 2.6 Mb PAR region of homology between the overwise largely nonhomologous X and Y sex chromosomes. These sex chromosomes mis-segregate more frequently than autosomes, giving aneuploidies that underlie e.g. Klinefelter or Turner syndromes (Shi et al. 2001; Hall et al. 2006). Nonetheless, their nondisjunction still remains relatively rare as compared to that expected from the short length of shared homology, suggesting the existence of mechanisms that ensure the presence of at least one CO in the PAR region. In fact, it was shown that PAR recombination and pairing are under different control from autosomes (Kauppi et al. 2012; Acquaviva et al. 2020). There are 10 – 20 fold more DSBs per Mb in the PAR region than in other chromosomal regions, which should dramatically favor the probability of an obligatory CO. In correspondence to this effect: (a) chromosome axis length, relative to DNA content is ten-fold longer when compared to the non-homologous X and Y portions and chromatin loops are correspondingly shorter; and (b) the PAR region is enriched for the RMM components (Rec114, Mei4, Mer2) required for genome-wide DSB formation. In addition, the XY pair exhibit different dynamics than autosomes, with delays in the prominence of RAD51 and DMC1 foci, pairing, and synapsis relative to occurrence of those events on the autosomes, perhaps because partner identification will be simpler of autosomes are already substantially paired.
3. Short chromosomes. Since DSB frequencies are normally proportional to axis length, shorter chromosomes should (all other things be equal) tend to have shorter axes and thus fewer DSBs. Short chromosomes should, therefore, be intrinsically more challenged for pairing and more likely to be CO-free than longer chromosomes. Indeed, the frequency of zero-CO chromosomes is generally higher for shorter chromosomes than longer ones e.g. in budding yeast (Kaback et al. 1992) and in human for the small chromosome 21 (Wang et al. 2017). This is especially true in nuclei with lower numbers of total COs, in accord with the fact that such nuclei tend to have shorter axes on all chromosomes (Wang et al. 2019).

Studies in budding yeast further suggest that these challenges would be even worse were it not for the existence of mechanisms that specifically enhance CO formation on short chromosomes. First, small chromosomes have more DSBs and a higher number of COs per physical length than longer chromosomes (Kaback et al. 1992; Mautino et al. 1993; Thacker et al. 2014; Lam and Keeney 2015). Second, recent data show that this risk is attenuated by several mechanisms which together give what is called the “short-chromosome boost” (Murakami et al. 2021). (1) Small chromosomes have larger axis/DNA ratio than longer chromosomes, i.e. longer axes and shorter loops, and thus more DSBs. (2) DSB proteins Rec114 and Mer2 plus the axis components Red1 and Hop1 (but not cohesin Rec8), are overrepresented on small chromosomes versus other chromosomes. Moreover, analysis of fusion chromosomes shows that this tendency is genetically encoded, rather than resulting from measuring of length per se (Murakami et al. 2021). Moreover, higher GC composition (which is known to favor both early replication and DSB formation (Costantini and Bernardi 2008; Blat et al. 2002) could be a contributing factor. (3) Rec114 and Mer2 are released later in pachytene than on longer chromosomes, thereby potentially allowing for increased numbers of DSBs (Murakami et al. 2021).

(4) Crossover homeostasis. Strikingly, when the number of total DSBs is directly reduced by genetic mutations in the *SPO11* gene, the number of COs does not decrease commensurately. This effect, referred to as "crossover homeostasis", is seen in budding yeast, *C. elegans,* and mouse (Martini et al. 2006; Rosu et al. 2011; Cole et al. 2012; Yokoo et al. 2012). In budding yeast, this effect is quantitatively attributable to the interplay of DSBs and CO interference: when the density of DSBs is reduced, any given DSB will have a reduced probability to be subjected to interference emanating from adjacent DSBs (Martini et al. 2006; Zhang et al. 2014b; Wang et al. 2015). In mouse, homeostatic control is also observed with respect to formation of early intermediates, and thus likely due to the effects of CO interference; however, an additional effect, as yet undefined, comes into play during CO maturation (Cole et al. 2012).

Crossover homeostasis as exerted on early recombination ameliorates the effects of DSB reduction on total COs but, because this effect depends on CO interference, it cannot affect the probability of occurrence of the obligatory CO. Perhaps effects that become apparent at later stages reflect additional feature that addresses this potential deficit.

Interestingly, in *C. elegans*, when programmed DSBs are eliminated by a *spo11* mutation, a single exogenously provided DSB is sufficient to ensure the obligatory CO (Altendorfer et al. 2020). Perhaps the normal parameters for precursor/CO designation are intrinsically robust in this organism; alternatively, these features might be enhanced by the single DSB in a condition-specific buffering process. In contrast, in *D. melanogaster*, a reduction in DSBs significantly compromises occurrence of the obligatory CO (Mehrotra and McKim 2006). This organism may not have acquired a buffering mechanism because it has specific backup mechanisms for segregating zero-crossover bivalents (Hawley and Theurkauf 1993).

In another interesting case, mouse meiosis appears to buffer itself against loss of the DSB-promoting hot spot factor PRDM9, at least over evolutionary time, by ready availability of a PRDM9-independent DSB mechanism (Powers et al. 2020).

#### 2. When DSB formation is normal but DSB-mediated pairing is defective

Defective pairing will reduce the number of precursor interactions available for CO designation, specifically in the unpaired regions, even if DSB formation occurs efficiently. In several such situations, region-limited pairing defects result either in maintenance of, or an increase in, the total number of COs that occur in remaining successfully paired regions. The overall outcome of these effects is predicted to be an increase in the probability that a first (obligatory) CO will occur in limited regions of the affected chromosome that are still normally paired,

In *A. thaliana* and *S. macrospora*, mutations that reduce homolog coalignment nonetheless exhibit almost wild-type levels of COs; and these COs occur exclusively in regions where SC has formed, implying that they arose in regions where coalignment-dependent precursors were present (Tessé et al. 2017; Duroc et al. 2014; Jahns et al. 2014). This phenomenon again suggests the existence of an effect which ensures occurrence of the obligatory CO even despite the limited proportion of coaligned regions were CO designation could occur.

#### 3. Efficient segregation of achiasmate chromosomes

While the canonical pathway for homolog segregation relies on the role of chiasmata to hold and orient chromosomes on the meiosis I spindle, some organisms have developed mechanisms that promote segregation of chromosomes in the absence of any CO.

In a few studied organisms, one sex lacks recombination altogether. The "achiasmate" sex is usually the one specified by two different types of sex chromosomes (e.g. XY versus XX) perhaps in response to the special challenge of their pairing/crossover formation. Up to now, no known organisms lack meiotic recombination altogether, presumably because the evolutionary importance of recombination. Notably, *D. melanogaster* male meiosis has neither recombination nor an SC. Instead, an interhomolog connection is provided by an assembly of four proteins: SNM (Stromalin in meiosis), MNM (modifier of mdg4) and UNO (univalent only), assisted by TEF (teflon; that recruits MNM). During prophase, this SUM complex of proteins forms variable foci that coalesce into a single bright focus when chromosomes condense, after which Anaphase I segregation is licensed by separase cleavage of UNO (Adams et al. 2020; Kabakci et al. 2022). And in the silk worm (*Bombyx mori*), female meiosis lacks COs/chiasmata but still has an SC-related structure that links homologs from prophase into Anaphase I, again providing the necessary connection (above, Xiang et al. 2024).

In other cases, special mechanisms exist to rescue chromosomes that fail to acquire even the "obligatory" crossover by chance (so-called "E0" chromosomes) or lack COs as an intrinsic chromosome-specific feature. For example, in *D. melanogaster* female meiosis, chiasmata direct the segregation of most chromosomes, but a second system (the "achiasmate" or "distributive" system) is used to segregate E0 chromosomes, the tiny 4th chromosomes that naturally lack COs, and, in some situations, nonhomologous chromosomes. In this species, peri-centric heterochromatin linkages link segregating chromosomes to achieve biorientation on the spindle via unique dynamic movements, and connections likely released by Topoisomerease II (Hawley and Theurkauf 1993; Fellmeth and McKim 2022; Hughes et al. 2018). Similar dynamic movements underlie regular achiasmate segregation in *Mesostoma ehrenbergii* (Oakley 1985). A backup mechanism for segregating achiasmate chromosomes also occurs in budding yeast, again involving centromere connections, here mediated by a specialized role of SC component Zip1 (Kurdzo et al. 2018); and heterochromatic interactions at centromeres are suggested analogously to mediate achiasmate segregation in mouse (Eyster et al. 2019).

### The evolutionary *raison d'être* of crossing-over is genetic shuffling

How has the meiotic recombination process been shaped so as to influence its evolutionary roles and so as to ensure the robustness of its mechanical roles? And how did the meiotic process evolve? Crossovers are a central feature of the standard meiotic program for both evolutionary and mechanistic reasons. The evolutionary dictate is likely the more important, since the mechanistic requirement for homolog connectedness can be achieved via other features (e.g. by remodeling SC components in Bivalent bridges as a glue between homologs like in silk worm females, Xiang et al. 2024 and discussion therein).

Genetic recombination contributes to "genetic shuffling". It is well established that, under appropriate circumstances, the creation of individuals with new combinations of alleles can enhance the ability of an organism to thrive in its environment, by several distinct effects (e.g. Otto 2009). It can be noted that genetic shuffling is achieved not only by CO but because maternal and paternal homologs of different chromosomes segregate independently at the first division (Mendelian "independent assortment" (IA)). In fact, the contribution of IA far outweighs that of genetic recombination. A recent quantitative analysis demonstrated that, for human male and female meiosis, the contribution of IA is ~ 30 times greater than that of CO (Veller et al. 2019). Nonetheless, crossing-over still makes a critical contribution because it produces *intra*-chromosomal genetic shuffling whereas IA produces only *inter*-chromosomal shuffling. [Note: confusingly, the population genetics community sometimes refers to both sources of genetic shuffling as "recombination"]. A related question is the evolutionary rationale for the existence of CO interference. Interference might be preserved for a mechanical reason, e.g. because it allows chromosome compaction at the prophase/metaphase (Darlington 1937); or might alternatively be important for the evolutionary role of COs. Three lines of evidence suggest that genetic recombination with CO exists because of the advantages it confers for genetic shuffling.

First, the two mechanistic functions of recombination, pairing and homolog disjunction, can both be ensured by mechanisms that do not involve recombination (above).

Second, CO is tightly regulated with regard to number (usually one or a few per chromosome; e.g. Mercier et al. 2015), spacing (usually evenly-spaced due to interference), and localization (which varies dramatically among organisms and even between sexes). None of these features is essential for fertility, apart from the imposition of the obligate chiasma (or CO). In *Arabidopsis thaliana,* a dramatic increase in the number of COs, with concomitant abrogation of interference, has no detectable effect on gamete viability (e.g. Mieulet et al. 2018); and since localization patterns are highly variable among different organisms, no specific pattern is critical for the mechanics of meiosis.

Third, in contrast, alterations in all three features are known to have a major impact on the contributions of recombination to evolution. Most prominently, too few COs will weaken the positive benefits whereas an excess of COs will be deleterious because it will increase the probability of breaking up existing good allele combinations. Localization patterns also have important effects on the amount of genetic shuffling (Veller et al. 2019). Finally, and most interestingly, even spacing of COs has recently been shown to increase the effectiveness of crossing-over for genetic shuffling (Veller et al. 2019). This finding provides the first documented evolutionary rationale for the existence of CO interference (and it is based on the effects of interference on emergence of new allele combinations, not on a role for chiasmata in the mechanics of meiosis).

### Modulation of CO levels for evolutionary adaptation

The number of COs per nucleus is an important parameter for the evolutionary fitness of an organism. One important consideration is that COs promote both diversification and conservation of allele combinations. These two effects trade off against one another, differently in different situations. In a situation where the fitness of an organism progressively increases in a fixed environment, CO levels are presumably set to occur at the particular level that will be most favorable. However, a different challenge arises when the environment fluctuates. Recent studies of meiosis provide new insights into how organisms deal with such situations.

#### 1. Modulation of CO levels via axis length changes

Several studies strongly support the important proposition that modulation of axis length represents a powerful, yet simple mechanism for modulation of CO levels to meet the dictates of evolution (Song et al. 2021). This mechanism is manifested in several different phenomena.

- **Per-nucleus covariation of crossovers across chromosomes**. The number of COs covaries across chromosomes on a per-nucleus basis, an effect achieved by global per-nucleus variation in chromosome axis lengths (Wang et al. 2019 and references therein). The outcome of such covariation is that the total number of COs per nucleus varies much more widely than would otherwise be the case, resulting in an increased frequency of "hyper-crossover" and "hypo-crossover" gametes. The increased availability of these two types of gametes, in turn, allows the organism to "hedge its bets" against fluctuations in its environment. When the environment remains the same as the one to which the organism has adapted, new allele combinations tend to be deleterious and hypo-crossover gametes will be favored. When the environment changes, it will be advantageous to have gametes with a higher level of recombination, because CO generates new (favorable) combinations faster than de novo mutation. Mathematical simulations confirm that per nucleus covariation confers a selective advantage when the environment involves alternating periods of stasis and change (Wang et al. 2019).
- **Different crossover levels in different sexes**—another form of gametic bet-hedging? Average per-nucleus CO numbers differ between male and female meiosis in a number of organisms. For example, CO levels are ~ 50% higher in female in human and mouse and ~ 70% higher in male than female in *Arabidopsis thaliana*. These gender-specific differences are directly attributable to differences in axis length (Wang et al. 2017). In extreme cases, COs are present in one sex but totally absent in the other. For example, in *D. melanogaster* and *B. mori*, COs occur only in female and male respectively (review in Zickler and Kleckner 1999; Hawley 2002). In fact, when achiasmate meiosis occurs, it is present in only one sex, as expected from the selective advantage conferred by CO. Gender-specific differences could represent another form of gametic bet-hedging, with higher- and lower- (or zero) CO gametes provided by the two sexes. This effect will be superimposed on, and thus further exaggerate, the bet-hedging effects of per nucleus covariation.
- **Adjustment of average crossover levels to different environmental conditions.** A given organism may have different CO levels in different environments. For example, geographically and ecologically diverse accessions of *A. thaliana* show differences in their number of chiasmata (Sanchez-Moran et al. 2002). In addition, recombination levels can change in response to temperature changes. This was originally shown for *D. melanogaster* (reviewed in Pazhayam et al. 2021). Also, several plants exhibit elevated CO frequencies at slightly higher temperature than in their “normal” growth conditions (Morgan et al. 2017) and in other plants, temperature shifts (from 22 °C to 30 °C in barley) cause a reduction in chiasmata and an alteration in CO distribution to a more interstitial location (Higgins et al. 2012; Phillips et al. 2015; Lloyd et al. 2018). In several of these cases (but not universally), chromosome axis (SC) lengths vary coordinately with CO frequencies, in accord with the cause-and-effect relationship emphasized above. All such effects should be synergistic with the beg-hedging effects of per-nucleus covariation and male/female differences in allowing organisms to adapt to changing environments.

#### 2. B-chromosomes as modulators of crossover frequency

One of the most intriguing phenomena linked to variations in CO levels concerns B chromosomes (discovered more than a century ago by Wilson 1907). These chromosomes are found across a large variety of plant, animal and fungal taxa (e.g. Houben et al. 2019). New sequencing methods reveal that B chromosomes can contain telomere and centromere sequences and protein-coding genes that are actively transcribed (Hanlon and Hawley 2018; Ruiz-Ruano et al. 2019). For example, in rye they contain Argonaute-like genes, SHOC1, PCH2 and SCC3, known to be involved in meiosis (Ma et al. 2017). They originate in some way from A chromosome fragments plus exhibit an accumulation of various repeated DNA and transposable elements (Ahmad et al. 2020).

B chromosomes occur in even or odd numbers ranging from 1 to 8 in rye (Jones and Rees 1982); do not recombine with standard (A) chromosomes; and show non-Mendelian inheritance (Jones et al. 2008; Harper et al. 2018; Hanlon and Hawley 2018). During meiotic prophase of *Crepis capillaris* and rye, they load ASY1/Hop1 on their axes slightly later than the A chromosomes and the SC component Zip1 assembles between Bs that are in even number or between folded-back single Bs or form multivalents when in odd numbers (Jones et al. 1989; Hesse et al. 2019).

Although their *modus operandi* on recombination remains unknown, these supernumerary chromosomes have been shown to influence recombination in several organisms. For example, when in the grasshopper *Eyprepocnemis plorans*, two Bs are present, they form a monochiasmate bivalent and this condition is correlated with a reduction of the number of chiasmata in the A chromosomes, suggesting a competition between the two (Henriques-Gil et al. 1982). In the ryegrass *Lolium perenne* the average chiasma frequency is reduced when two B chromosomes are present when compared to plants with zero B chromosomes (10.9 *versus* 12.1 per cell; Harper et al. 2018). However, in other cases the presence of B chromosomes is associated with an overall increase in chiasma frequencies (Thomson et al. 1984; Jauhar and Crane 1990). B chromosomes also often impact on the distribution of crossovers along the homologs. For example, redistribution of chiasmata from distal to interstitial locations in presence of B chromosomes is found in ryegrass, rye and in the wheat *Triticum speltoides* (Jones and Rees 1967; Zarchi et al. 1974; Harper et al. 2018). Although B chromosomes can also diminish the viability of the organism carrying them (e.g. Parker et al. 1991), the fact that they can modulate both CO numbers and their redistribution along homologs could explain why B chromosomes are observed in several taxa through evolution: they could be an interesting and beneficial way for modulating the impact of COs on allele shuffling. Possible roles for genetic engineering in plants have also been discussed (Jones and Ruban 2019).

#### 3. Crossover maturation inefficiency and the maternal age effect in human female meiosis

Quantitative analysis of human male and female meiosis has not only provided rigorous documentation of how and why (differences in) axis length in the two sexes determines (differences in) CO frequencies (above) but also revealed that female meiosis is specifically afflicted by a unique defect: an apparently random subset of crossover-designation interactions fail to mature to COs (Wang et al. 2017). This CO maturation inefficiency (CMI) explains why the basal rate of aneuploidy is substantially higher for gametes from mothers of normal child-bearing age than for paternal gametes, despite the fact that the overall CO frequency is higher in female than in male (above). CMI has this effect for two reasons. First, it substantially increases the frequency of zero-crossover chromosomes and thus mis-segregation of homologs. Second, equally importantly, CMI alters the distributions of COs along the chromosomes in a way predicted to compromise tension-dependent bipolar orientation on the meiosis I spindle (Wang et al. 2017).

Female meiosis is also famously characterized by a dramatic increase in oocyte aneuploidy with increasing maternal age (Gruhn et al. 2019; Wartosch et al. 2021; Gruhn and Hoffmann 2022). This effect cannot be explained directly by CMI because the age-dependent delay occurs after pachytene, and thus after CO formation is completed. However, the effects of CMI could synergize with age-dependent effects, e.g. on sister cohesion or spindle function, as an underlying, potentially critical, sensitizing factor.

From an evolutionary perspective, it is interesting to consider whether elevated female aneuploidy, during and/or after normal reproductive age, might have an evolutionary benefit (e.g. to prevent births in mothers who are too old to raise their children or to provide non-reproducing "grandmothers") or is simply a happenstance that selective pressure has not yet eliminated (discussion in Wang et al. 2017; Gruhn and Hoffmann 2022).

#### 4. Evolution of stable autopolyploidy

When a diploid organism undergoes whole genome duplication, the outcome is an "autotetraploid" whose genome comprises four identical or nearly-identical copies of each chromosome. As might be expected, a newly-arisen autotetraploid exhibits aberrant pairing and CO formation, promiscuously among the four homologs. However, stable autopolyploid lines evolve in which, by metaphase I, homologs are connected only pairwise, leading to regular segregation and high levels of fertility. How this is achieved is unknown. It has recently been shown, for *Arabidopsis arenosa*, that CO interference is defective in a newly-formed autotetraploid and robust in an evolved autotetraploid (Morgan et al. 2021). It is proposed that the key event is evolution of a "supercharged" CO interference process which can operate effectively on multivalent coalignment configurations. Such an effect, in combination with a sufficiently long "interference distance", would have the desired result. In accord with this possibility, when an evolved autotetraploid undergoes a further whole genome duplication, the resulting auto-octoploid is immediately fertile, with fully regular pairwise crossover connections at meiosis I, and thus without the need for further evolutionary change (Morgan et al. 2021). This outcome is directly explained by supercharged CO interference, which will be insensitive to the number of coaligned copies, and is more difficult to explain by mechanisms that achieve pairwise interactions during the coalignment stage.

## Speciation

Many hypotheses for the basis of speciation have been discussed. Among these was the idea that the fundamental effect was rejection of CO recombination interactions in response to base pair mismatches (Hunter et al. 1996). This hypothesis has recently received strong support from analysis of an inter-specific yeast hybrid in which the genome sequences differ by 12%. Meiosis-specific repression of the mismatch repair-specific component Msh2 or the anti-recombination factor Sgs1 results in a 70-fold increase in hybrid fertility, resulting normal levels of viable gametes (Bozdag et al. 2021).

Interestingly, in mouse and human, speciation is thought to involve the DSB-promoting zinc finger protein PRDM9. In mice that carry two PRDM9 alleles with different binding specificities, fertility correlates with the extent to which an allele binds both homologs at the DSB site: it controls via an effect which, thus, appears to be downstream of the DSB process (Davies et al. 2021; Paigen and Petkov 2018). This effect might involve DSB-mediated coalignment, which will involve nascent strand invasion of the DSB on the partner.

### How did meiosis evolve?

Many speculations have been presented regarding the path by which the meiotic chromosomal program (and thereby efficient sexual reproduction) might have evolved (e.g. Wilkins and Holliday 2009; Lenormand et al. 2016). The considerations presented above suggest that the central feature of this program is direct physical and functional coupling of DNA events of recombination, which clearly have evolved from mitotic recombinational repair of DSBs, with changes in global chromosome state that appear to be derived by modulation of the latter stages of the mitotic chromosome cycle (Zickler and Kleckner 2023). Thus, the key event in evolution of meiosis should be linkage of recombination complex association with developing or developed chromosome structural axes.

In this regard, it can be noted that at some low level, mitotic recombinational DSB repair can occur between homologs (rather than the usual sister chromatids) to give COs, that inter-homolog DNA COs that arise by recombination during the mitotic program can direct homolog segregation to opposite poles; and that occurrence of a DSB during the mitotic program results in local accumulation of cohesins, which are prominent features of mitotic and meiotic chromosome axes, at the site of DSB repair. Furthermore, the decision as to whether a DSB selects either a sister partner or a homolog partner appears to involve direct interplay between recombination proteins and cohesins (Hong et al. 2019). Overall, it is not difficult to imagine a path from recombination protein/cohesin ensembles to association of recombination complexes with later-stage cohesin-containing axes as the key event which nucleated development of the meiotic program.

More recently, it has been proposed that axis-association of recombination complexes to axial structural features has allowed evolution by coupling of the interhomolog interaction program to basic events of mitotic chromosome morphogenesis (Zickler and Kleckner 2023).

### Looking ahead

Meiosis research continues to expand in all directions with each finding necessarily leading to new questions. Nonetheless, despite tremendous progress since the discovery of meiosis, several fundamental basic problems remain unsolved. For example, with regard to the mechanics of meiosis, it is still difficult to understand how many different long, thin chromosomes packed into a crowded nucleus manage to find each other and come together in space into paired/synapsed units without creation of a tangled mess. Furthermore, the mechanism of CO patterning, with its two basic features of the obligatory CO and CO interference, remains to be solved. In these contexts, a key unique feature of meiosis is that recombination complexes are physically and functionally linked to chromosome structures (axes/SCs) at every stage, with communication in both directions. An important step will be understanding the roles of this association for both homolog pairing and crossover patterning. This will include an understanding of the role(s) of the SC, the most famous and prominent structure of meiosis which, nonetheless, is absent in some organisms. And since the meiotic program of interhomolog interactions occurs after chromosome replication, an intrinsic requirement is that key events occur between non-sister chromatids of homologs rather than between sisters.

More broadly, the many biological, evolutionary, medical and agricultural implications of meiosis, and how the relevant effects are modulated in different organisms and different situations, remain to be addressed. It is also very striking that, while early cytological studies covered a wide variety of organisms, molecular biology studies have followed the tradition of that field to focus more and more on a few model organisms. As one reviewer suggested to us: with increased genomic resources and sophisticated genome editing techniques, the next century could potentially bring us back to study meiosis on a molecular level in ciliates, grasshoppers, ferns or salamanders, uncovering new and unexpected insights into meiosis.
